# Supplementary material for: In Silico Selection and Evaluation of Pugnins with Antibacterial and Anticancer Activity Using Skin Transcriptome of Treefrog (Boana pugnax)
Source: Pharmaceutics. 2021 Apr 18;13(4):578. doi: 10.3390/pharmaceutics13040578 (PMC8074116; doi:10.3390/pharmaceutics13040578)
Supplement: Supplementary file 1 [file pharmaceutics-13-00578-s001.zip › pharmaceutics-1151638-supplementary.pdf]

pharmaceutics

MDPI

Supplementary Materials: In Silico Selection and Evaluation of Pugnins with Antibacterial and Anticancer Activity Using Skin Transcriptome of Treefrog (*Boana pugnax*)

Yamil Liscano, Laura Medina, Jose Oñate-Garzón, Fanny Gúzman, Monica Pickholz and Jean Paul Delgado

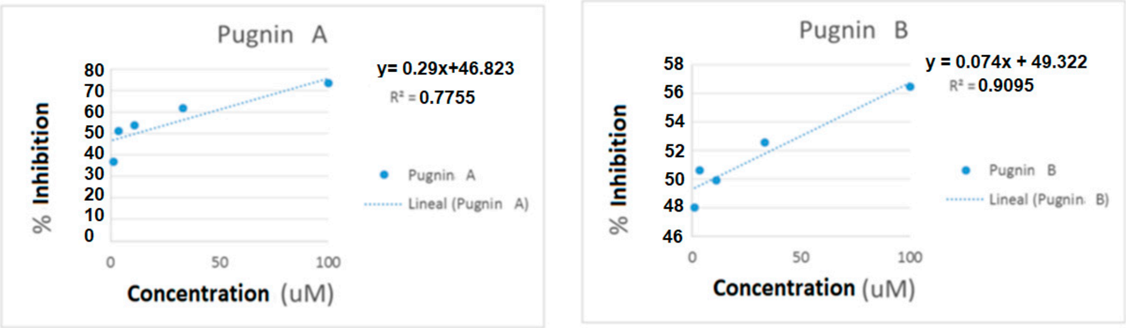

Figure S1. Percentage of growth inhibition of *S. aureus* of the pugnins A and B, with their respective R^2 of the concentration curve of each peptide and the equation of the line is also observed.

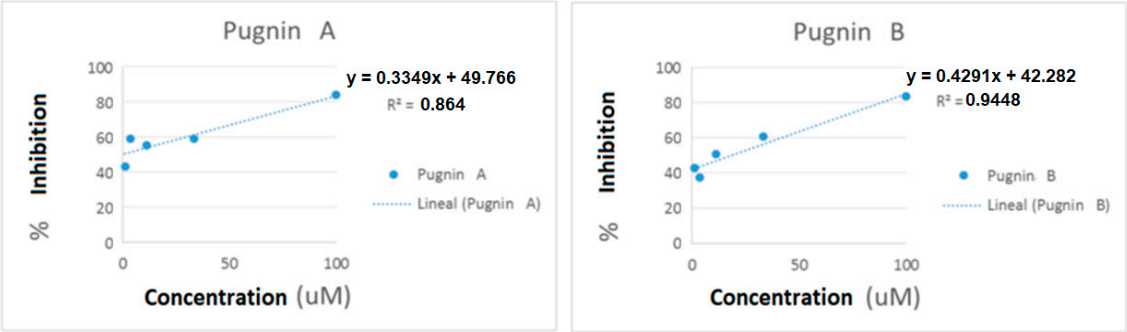

Figure S2. Percentage of growth inhibition of *E. faecalis* of the pugnins A and B, with their respective R^2 of the concentration curve of each peptide and the equation of the line is also observed.

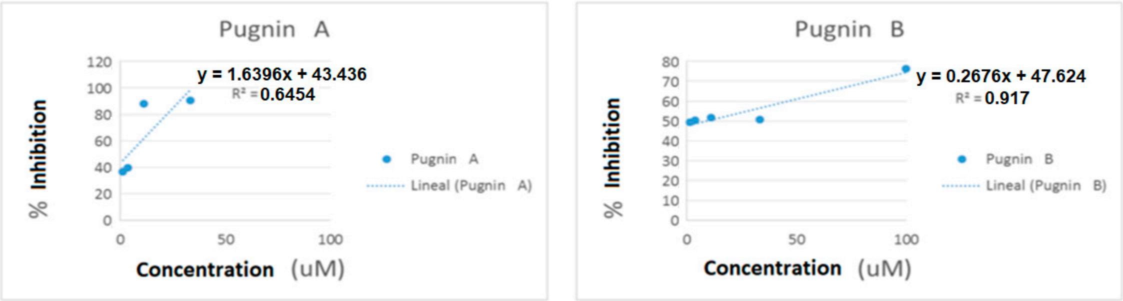

Figure S3. Percentage of growth inhibition of *P. aeruginosa* of the pugnins A and B, with their respective R^2 of the concentration curve of each peptide and the equation of the line is also observed.

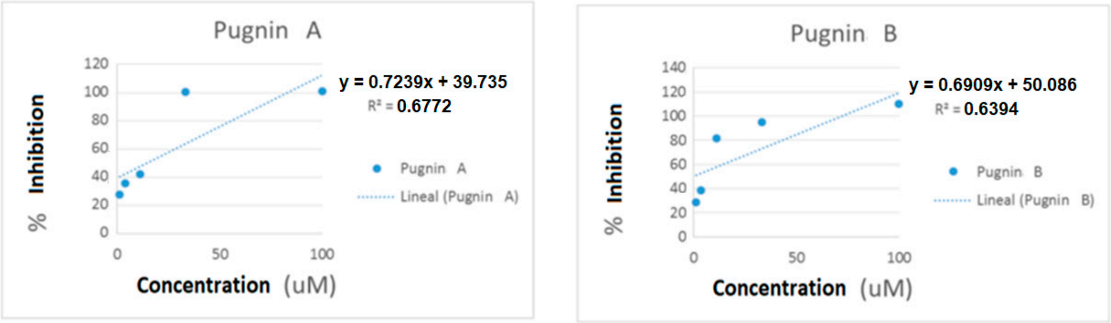

Figure S4. Percentage of growth inhibition of *E. coli* of the pugnins A and B, with their respective R^2 of the concentration curve of each peptide and the equation of the line is also observed.

##### Extended Information		
[Comments]		
Sample name	3665 TFE 30%	
Comment		
User		
Division		
Company	NBC PUCV	
[Detailed Information]		
Creation date	##########	
Data array type	Linear data array * 3	
Horizontal axis	Wavelength [nm]	
Vertical axis(1)	Mol. Ellip.	
Vertical axis(2)	HT [V]	
Vertical axis(3)	Abs	
Start	250 nm	
End	190 nm	
Data interval	0.5 nm	
Data points		121
[Measurement Information]		
Instrument name	DICROISMO PUCV	
Model name	J-815	
Serial No.	B052661168	
Accessory	CDF-426S	
Accessory S/N	B009861183	
Temperature	19.98 C	
Control sensor	Holder	
Monitor sensor	Holder	
Start Mode	Start immediately	
Cell length	10 mm	
Measurement date	##########	
Photometric mode	CD. HT. Abs	
Measure range	250 - 190 nm	
Data pitch	0.5 nm	
Sensitivity	Standard	
D.I.T.	2 sec	
Bandwidth	1.00 nm	
Start mode	Immediately	
Scanning speed	100 nm/min	
Baseline correction	None	
Shutter control	Manual	
CD detector	PMT	
PMT voltage	Auto	
Accumulations		3

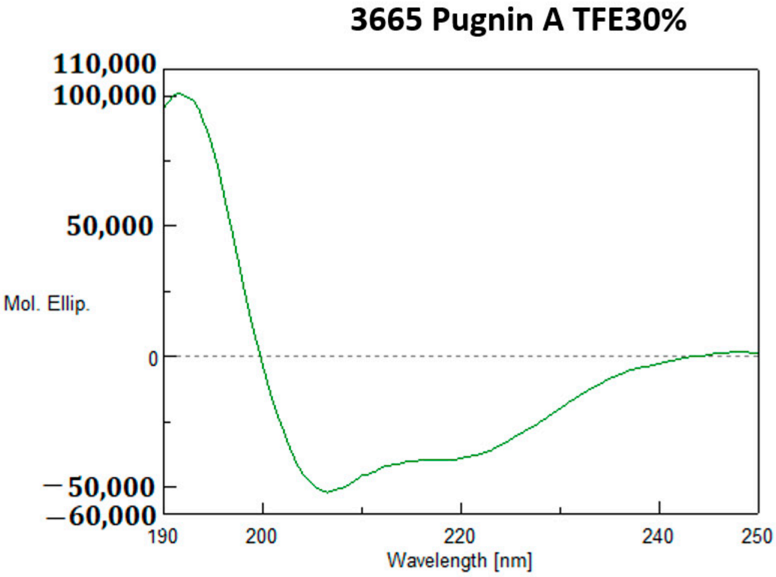

	3665 TFE 30%	3665 Agua	3666 TFE 30%	3666 Agua
Alfa	0.905	0.153	0.751	0.157
Beta	0.000	0.000	0.001	0.000
Turn	0.015	0.185	0.068	0.130
PP2	0.001	0.000	0.028	0.057
Unrd	0.079	0.662	0.152	0.656
RMSD	0.525	0.472	0.281	0.968
NRMSD	0.038	0.025	0.028	0.067
Base de datos	SP37A			
Algoritmo	Contin			

Figure S5. Circular dichroism of pugnin A.

##### Extended Information	
[Comments]	
Sample name	3666 TFE30%
Comment	
User	
Division	
Company	NBC PUCV
[Detailed Information]	
Creation date	##########
Data array type	Linear data array * 3
Horizontal axis	Wavelength [nm]
Vertical axis(1)	Mol. Ellip.
Vertical axis(2)	HT [V]
Vertical axis(3)	Abs
Start	250 nm
End	190 nm
Data interval	0.5 nm
Data points	121
[Measurement Information]	
Instrument name	DICROISMO PUCV
Model name	J-815
Serial No.	B052661168
Accessory	CDF-426S
Accessory S/N	B009861183
Temperature	19.98 C
Control sonsor	Holder
Monitor sensor	Holder
Start Mode	Start immediately
Cell length	10 mm
Measurement date	##########
Photometric mode	CD. HT. Abs
Measure range	250 - 190 nm
Data pitch	0.5 nm
Sensitivity	Standard
D.I.T.	2 sec
Bandwidth	1.00 nm
Start mode	Immediately
Scanning speed	100 nm/min
Baseline correction	None
Shutter control	Manual
CD detector	PMT
PMT voltage	Auto
Accumulations	3

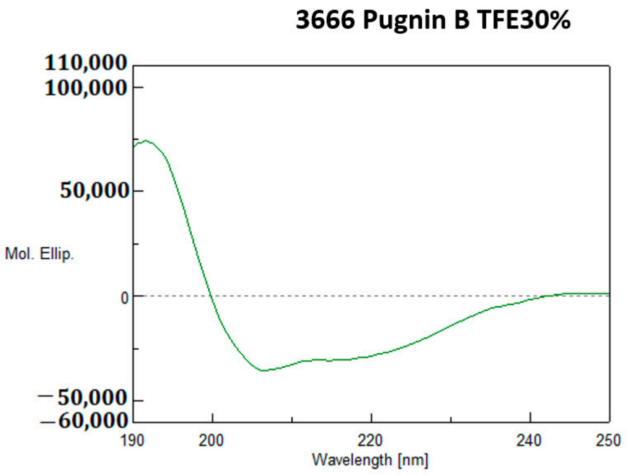

	3665 TFE 30%	3665 Agua	3666 TFE 30%	3666 Agua
Alfa	0.905	0.153	0.751	0.157
Beta	0.000	0.000	0.001	0.000
Turn	0.015	0.185	0.068	0.130
PP2	0.001	0.000	0.028	0.057
Unrd	0.079	0.662	0.152	0.656
RMSD	0.525	0.472	0.281	0.968
NRMSD	0.038	0.025	0.028	0.067
Base de datos	SP37A			
Algoritmo	Contin			

Figure S6. Circular dichroism of pugnin B.

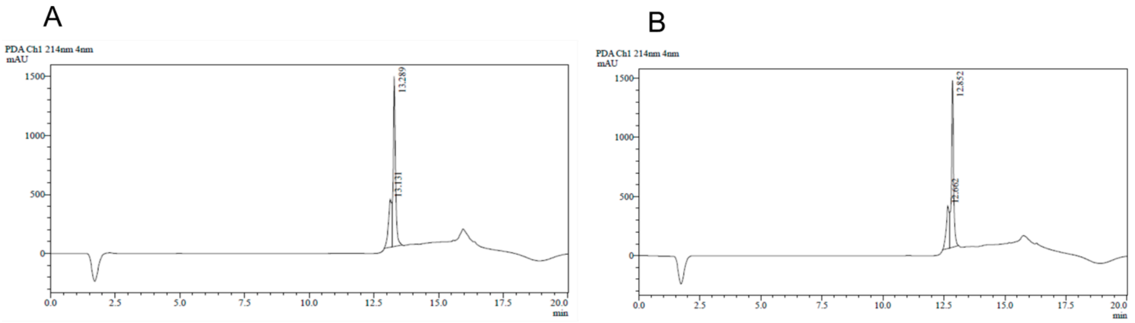

Figure S7. Chromatograms of pugnins. (**A**) Pugnin A. (**B**) Pugnin B.

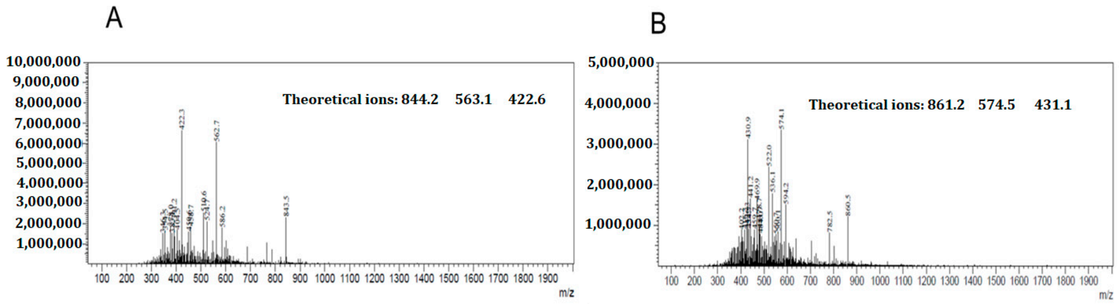

Figure S8. Mass spectrometry of pugnins. (**A**) Pugnin A. (**B**) Pugnin B.

Table S1. Two-way ANOVA of antibacterial activity of pugnins on *E.coli*. * significant value; ** high significant value; **** very high significant value.

Table Analyzed	Data 1	*P-Value*	*P* Value Summary	Significant?	*P-Value*
Two-way ANOVA	Ordinary				
Alpha	0,05				
Source of Variation	% of total variation	*P* value	*P* value summary	Significant?	
Interaction	10,22	< 0,0001	****	Yes	
Row Factor	81,01	< 0,0001	****	Yes	
Column Factor	8,772	< 0,0001	****	Yes	
ANOVA table	SS	DF	MS	F (DFn, DFd)	*P* value
Interaction	3905	8	488,2	F (8, 30) = 255137	*P* < 0,0001
Row Factor	30948	4	7737	F (4, 30) = 4,044e+006	*P* < 0,0001
Column Factor	3351	2	1676	F (2, 30) = 875722	*P* < 0,0001
Residual	0,0574	30	0,001913		
Number of missing values	0				

Table S2. Two-way ANOVA of antibacterial activity of pugnins on *P. aeruginosa.* * significant value; ** high significant value; **** very high significant value.

Table Analyzed	Data 1	*P-Value*	*P* Value Summary	Significant?	*P-Value*
Two-way ANOVA	Ordinary				
Alpha	0,05				
Source of Variation	% of total variation	*P* value	*P* value summary	Significant?	
Interaction	30,27	< 0,0001	****	Yes	
Row Factor	61,02	< 0,0001	****	Yes	
Column Factor	8,710	< 0,0001	****	Yes	
ANOVA table	SS	DF	MS	F (DFn, DFd)	*P* value
Interaction	5169	8	646,1	F (8, 30) = 136695	*P* < 0,0001
Row Factor	10419	4	2605	F (4, 30) = 551067	*P* < 0,0001
Column Factor	1487	2	743,6	F (2, 30) = 157320	*P* < 0,0001
Residual	0,1418	30	0,004727		
Number of missing values	0				

Table S3. Two-way ANOVA of antibacterial activity of pugnins on *E. faecalis.* * significant value; ** high significant value; **** very high significant value.

Table Analyzed	Data 1	*P-Value*	*P* Value Summary	Significant?	*P-Value*
Two-way ANOVA	Ordinary				
Alpha	0,05				
Source of Variation	% of total variation	*P* value	*P* value summary	Significant?	
Interaction	12,46	< 0,0001	****	Yes	
Row Factor	71,67	< 0,0001	****	Yes	
Column Factor	15,87	< 0,0001	****	Yes	
ANOVA table	SS	DF	MS	F (DFn, DFd)	*P* value
Interaction	1045	8	130,6	F (8, 30) = 34361	*P* < 0,0001
Row Factor	6007	4	1502	F (4, 30) = 395190	*P* < 0,0001
Column Factor	1330	2	664,9	F (2, 30) = 174971	*P* < 0,0001
Residual	0,1140	30	0,0038		
Number of missing values	0				

Table S4. Two-way ANOVA of antibacterial activity of pugnins on *S. aureus.* * significant value; ** high significant value; **** very high significant value.

Table Analyzed	Data 1	*P-value*	*P* Value Summary	Significant?	*P-Value*
Two-way ANOVA	Ordinary				
Alpha	0,05				
Source of Variation	% of total variation	*P* value	*P* value summary	Significant?	
Interaction	24,22	< 0,0001	****	Yes	
Row Factor	18,57	< 0,0001	****	Yes	
Column Factor	57,21	< 0,0001	****	Yes	
ANOVA table	SS	DF	MS	F (DFn, DFd)	*P* value
Interaction	1389	8	173,7	F (8, 30) = 35492	*P* < 0,0001
Row Factor	1065	4	266,3	F (4, 30) = 54431	*P* < 0,0001
Column Factor	3282	2	1641	F (2, 30) = 335401	*P* < 0,0001
Residual	0,1468	30	0,004893		
Number of missing values	0				

Table S5. Two-way ANOVA of Hemolytic activity of pugnins. * significant value; ** high significant value; **** very high significant value.

Table Analyzed	Data 1	*P-Value*	*P* Value Summary	Significant?	*P-Value*
Two-way ANOVA	Ordinary				
Alpha	0,05				
Source of Variation	% of total variation	*P* value	*P* value summary	Significant?	
Interaction	17,86	< 0,0001	****	Yes	
Row Factor	56,70	< 0,0001	****	Yes	
Column Factor	25,43	< 0,0001	****	Yes	
ANOVA table	SS	DF	MS	F (DFn, DFd)	*P* value
Interaction	7586	8	948,2	F (8, 30) = 5985	*P* < 0,0001
Row Factor	24074	4	6019	F (4, 30) = 37991	*P* < 0,0001
Column Factor	10797	2	5398	F (2, 30) = 34077	*P* < 0,0001
Residual	4,753	30	0,1584		
Number of missing values	0				

Table S6. One-way ANOVA and post-HOC comparisons of control with MTT cytotoxicity treatments of pugnins in HACAT cells. * significant value; ** high significant value; **** very high significant value.

**Table Analyzed**			**One-Way ANOVA Data**	
**ANOVA Summary**				
F			47,68	
P value			<0,0001	
P value summary			****	
Are differences among means statistically significant? ($P < 0.05$)			Yes	
R square			0,7607	
Dunnett's multiple comparisons test	Mean Diff,	95% CI of diff,	Significant?	Summary
Control vs. Pugnina A	26,45	9,234 to 43,67	Yes	**
Control vs. Pugnina B	72,45	55,23 to 89,67	Yes	****

Table 7. One-way ANOVA and post-HOC comparisons of control with MTT cytotoxicity treatments of pugnins in MFC7 cells. * significant value; ** high significant value; **** very high significant value.

**Table Analyzed**			**One-Way ANOVA Data**	
**ANOVA Summary**				
F			321,5	
P value			<0,0001	
P value summary			****	
Are differences among means statistically significant? ($P < 0.05$)			Yes	
R square			0,9554	
Dunnett's multiple comparisons test	Mean Diff,	95% CI of diff,	Significant?	Summary
Control vs. Pugnina A	77,84	68,84 to 86,83	Yes	****
Control vs. Pugnina B	84,88	75,88 to 93,88	Yes	****

Table 8. One-way ANOVA and post-HOC comparisons of control with MTT cytotoxicity treatments of pugnins in PC3 cells. * significant value; ** high significant value; **** very high significant value.

**Table Analyzed**			**One-Way ANOVA Data**	
**ANOVA Summary**				
F			3,445	
P value			0,0450	
P value summary			*	
Are differences among means statistically significant? ($P < 0.05$)			Yes	
R square			0,1868	
Dunnett's multiple comparisons test	Mean Diff,	95% CI of diff,	Significant?	Summary
Control vs. Pugnina A	13,69	0,08111 to 27,31	Yes	*
Control vs. Pugnina B	−1,387	−15,00 to 12,23	No	ns
